# Neonatal Sepsis in Dogs Caused by *Beta-Hemolytic Streptococcus* Sourced From Maternal Mastitis—Case Report

**DOI:** 10.1155/crve/6551231

**Published:** 2025-12-11

**Authors:** Keylla Helena Nobre Pacífico Pereira, Júlia Cosenza Mendonça, Kárita da Mata Fuchs, Gleice Mendes Xavier, Diogo Ribeiro Câmara, Fabiana Ferreira Souza, Maria Lucia Gomes Lourenço

**Affiliations:** ^1^ Veterinary Neonatology Research Group, Department of Veterinary Clinics, School of Veterinary Medicine and Animal Science, São Paulo State University (UNESP), Botucatu, Brazil, usp.br; ^2^ Department of Veterinary Medicine, Federal University of Alagoas (UFAL), Viçosa, Brazil, ufal.edu.br; ^3^ Department of Veterinary Surgery and Animal Reproduction, School of Veterinary Medicine and Animal Science, São Paulo State University (UNESP), Botucatu, Brazil, usp.br

**Keywords:** bacterial infections, milk, mortality in dogs, puppies, SIRS

## Abstract

Bacterial infections are common during the neonatal period, and sepsis is one of the main causes of mortality in dogs in the first 21 days. Maternal clinical and subclinical mastitis is one of the main sources of neonatal infection, leading to the ingestion of milk with bacteria and bacterial toxins, known as toxic milk syndrome. The objective of this report was to describe a case of neonatal sepsis in puppies secondary to maternal clinical mastitis, as well as its diagnosis and treatment. Three 5‐day‐old Pug dogs presented signs of neonatal infection: diarrhea, body erythema, and omphalitis. The bitch had purulent content in all of her breasts. Leukopenia due to neutropenia and monocytosis was observed in the puppies′ blood count. The milk microbiological culture revealed *beta-hemolytic Streptococcus*, which was sensitive to antibiotics from the cephalosporin and penicillin classes according to the antibiogram. The puppies were separated from their mother, fed with breast milk substitute, and treated with ceftriaxone. The mother was treated with amoxicillin with potassium clavulanate. Neonatal and maternal infections were responsive to the antibiotics used, demonstrating the absence of signs of infection and blood counts without changes after 7 days of treatment. Early diagnosis and treatment were essential for the survival of the litter.

## 1. Introduction

The neonatal period in dogs is associated with high mortality rates, reaching up to 35% [1–5]. The main causes of death are associated with parturition, maternal and environmental factors, and infections [1, 6, 7]. Infectious diseases, especially bacterial, are the main causes of mortality in dogs during the first 3 weeks of life [8, 9] distributed among local infections (~18%) or systemic infections (~39%), that might evolve to sepsis, diagnosed in ~43.3% of puppies with bacterial infection [5].

Sepsis is characterized by host immune response dysregulation to generalized infection, leading to systemic inflammatory response syndrome (SIRS), which culminates in systemic vasodilation, tissue hypoperfusion, and cellular and organic dysfunction [10–12]. The incidence of neonatal sepsis in dogs is high, and approximately 15% of newborns can be affected. A high mortality rate of approximately 25% has been described in affected patients, especially when there is no early assistance [5].

The main bacterial agents involved in neonatal sepsis in dogs are *Escherichia coli*, *Staphylococcus* spp., *Streptococcus* spp., *Klebsiella* spp., *Enterococcus* spp., *Pseudomonas aeruginosa*, *Chlamydia psittaci*, *Ureaplasma* spp., *Proteus* spp., *Salmonella* spp., *Klebsiella* spp., *Mannheimia haemolytica*, *Pasteurella multocida*, and *Enterobacter* spp. [6–8].

The mother is the main source of infection and is responsible for approximately 88% of neonatal sepsis cases. The infection can be intrauterine, through the genital tract at the time of birth, through milk, and maternal oronasal secretions. Among these, clinical or subclinical maternal mastitis, which is commonly associated with the presence of bacteria and bacterial toxins in milk, can culminate in toxic milk syndrome, a condition characterized by gastrointestinal disorders, systemic infection, and progression to sepsis in neonates [5, 7].

The main clinical signs presented by neonates with sepsis are apathy, diarrhea, body erythema, omphalitis, abdominal hematomas, neonatal triad (hypothermia, hypoglycemia, and dehydration), and cyanosis or necrosis of the limb ends, tail, or ears [13]. The diagnosis of neonatal sepsis involves clinical evaluation and laboratory tests, such as blood count, bacterial culture, and antibiogram. Treatment should begin with the selection of broad‐spectrum antibiotics that are safe for newborns, such as amoxicillin with potassium clavulanate, ceftriaxone, or ceftiofur, as well as supportive therapy [5, 6]. However, a lack of clinical assessment of the mother and the litter is still common, resulting in a failed diagnosis or late treatment. The objective of this report was to describe a case of neonatal bacterial sepsis in puppies resulting from maternal clinical mastitis, as well as its diagnosis and treatment.

## 2. Case Presentation

**Figure 1:**
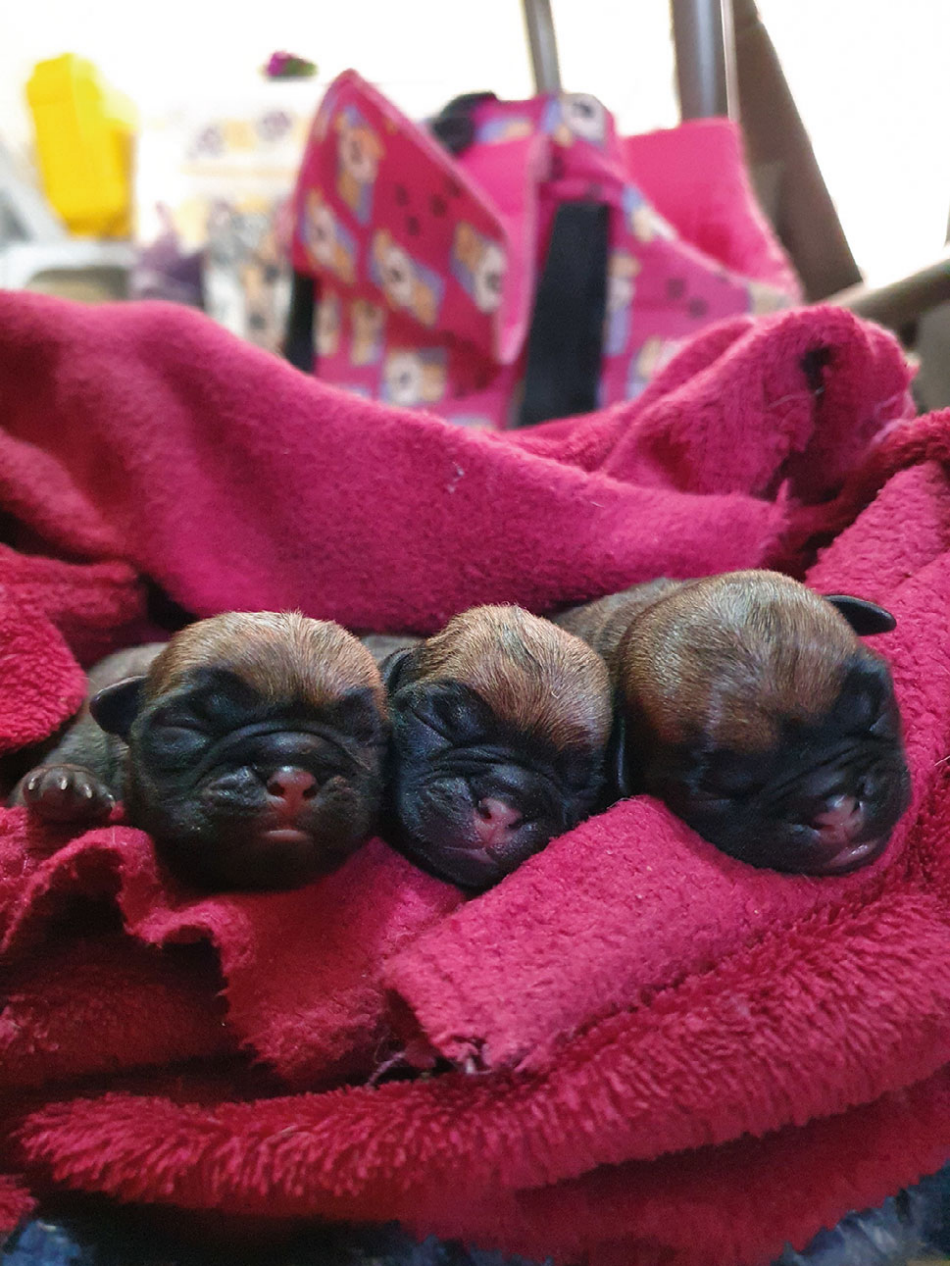
Neonatal Pug puppies.

A litter of three 5‐day‐old Pug dogs (Figure 1) with a history of diarrhea for 3 days was attended at FMVZ Unesp Veterinary Hospital, Botucatu, São Paulo. The puppies were born through an unassisted eutocic birth and, according to the owner, they were suckling normally from the bitch.

With respect to maternal information, she was pluriparous (third pregnancy), 6 years old, fed premium commercial food, vaccinated, dewormed, and had no history of disease or drug administration during pregnancy. No changes were reported in the previous litters. The dog was kept in a home environment, with access to a dirt yard, to which the owner reported that it was constantly lying down.

**Figure 2:**
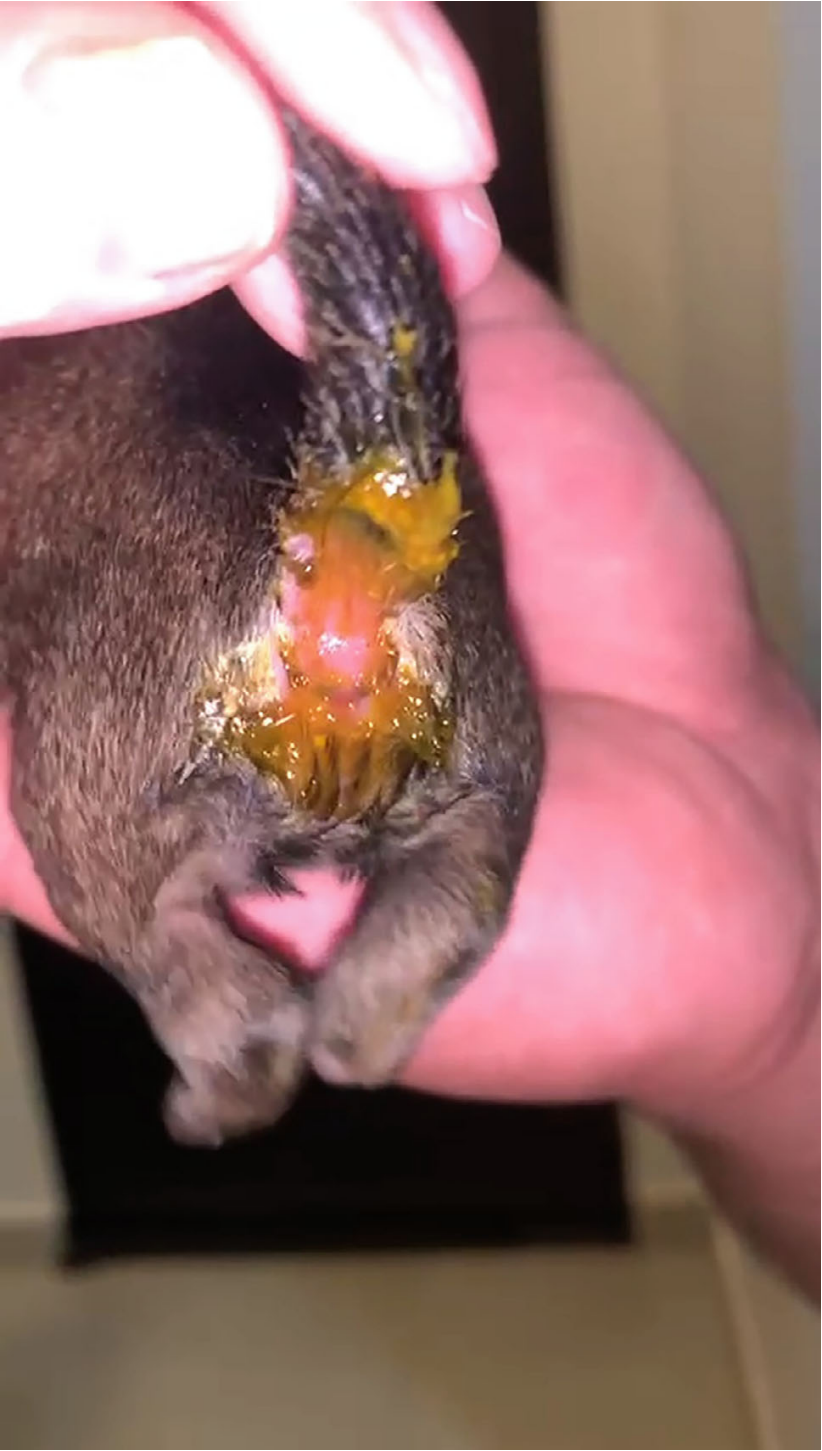
Diarrhea due to toxic milk syndrome in a neonatal puppy. The feces of a healthy newborn should be consistent and should not be pasty or diarrheal. Diarrhea is often associated with gastrointestinal disorders, such as bacterial, parasitic, or viral infections, and nutritional or management errors.

**Figure 3:**
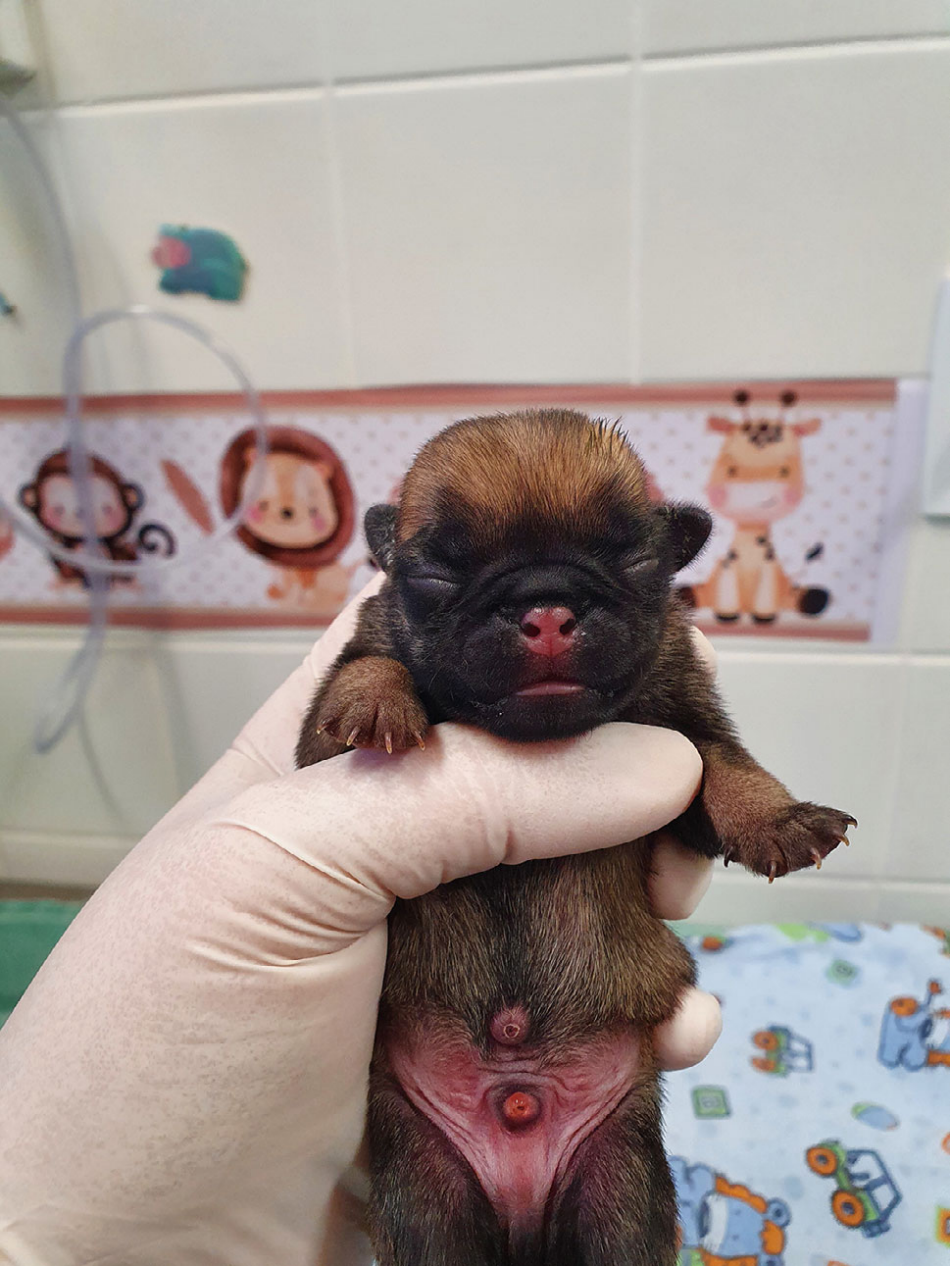
Abdominal erythema in the neonatal puppy. The abdomen of a healthy newborn is light pink in color. The observation of reddish (erythematous) coloration may strongly indicate systemic bacterial infection or neonatal sepsis. Abdominal erythema occurs primarily due to vasodilation caused by systemic inflammatory response syndrome.

On clinical examination (Table 1), the neonates had pink mucous membranes, an average heart rate (HR) of 218.6 beats per minute (bpm), an average respiratory rate (RR) of 36.6 movements per minute (mpm), a weak sucking reflex in one puppy, rooting and vestibular straightening present and strong in all puppies, an average body temperature of 36°C, an average blood glucose of 98 mg/dL, slight dehydration in one neonate (dark color of urine and hemoconcentration in the blood count), and an average weight of 212.3 g. Furthermore, they demonstrated clinical signs of neonatal infection, such as diarrhea (Figure 2), abdominal erythema (Figure 3), and omphalitis.

**Table 1:** Clinical parameters of newborn puppies.

**Parameters**	**Neonate 1**	**Neonate 2**	**Neonate 3**
HR	192 bpm	224 bpm	240 bpm
RR	30 mpm	44 mpm	30 mpm
Body temperature	35.2°C	36.8°C	36.2°C
Blood glucose	62 mg/dL	122 mg/dL	110 mg/dL
Weight	204 g	226 g	207 g

Abbreviations: bpm, beats per minute; g, grams; mpm, movements per minute.

Neonate 1 presented constant vocalization, a weak sucking reflex, a neonatal triad (hypothermia, hypoglycemia, and dehydration), and bradycardia. Immediate assistance was provided with warming in an incubator (32°C), oral 12.5% glucose replacement (with an orogastric tube, 0.5 mL/100 g of weight), and subcutaneous fluid therapy with lactated Ringer (2 mL/100 g of weight). After treatment, improvements in clinical parameters were observed, with an HR of 212 bpm, a body temperature of 36.6°C, a blood glucose level of 96 mg/dL, and a strong sucking reflex. After neonatal stabilization, additional tests, including blood counts and coproparasitology, were requested for all puppies. Blood counts revealed leukopenia (an average of 3.3 × 10^3^/*μ*L leukocytes), neutropenia (an average of 1.6 × 10^3^/*μ*L neutrophils), and monocytosis (an average of 2.8 × 10^3^/*μ*L monocytes). Coproparasitology was negative for the presence of endoparasites.

**Figure 4:**
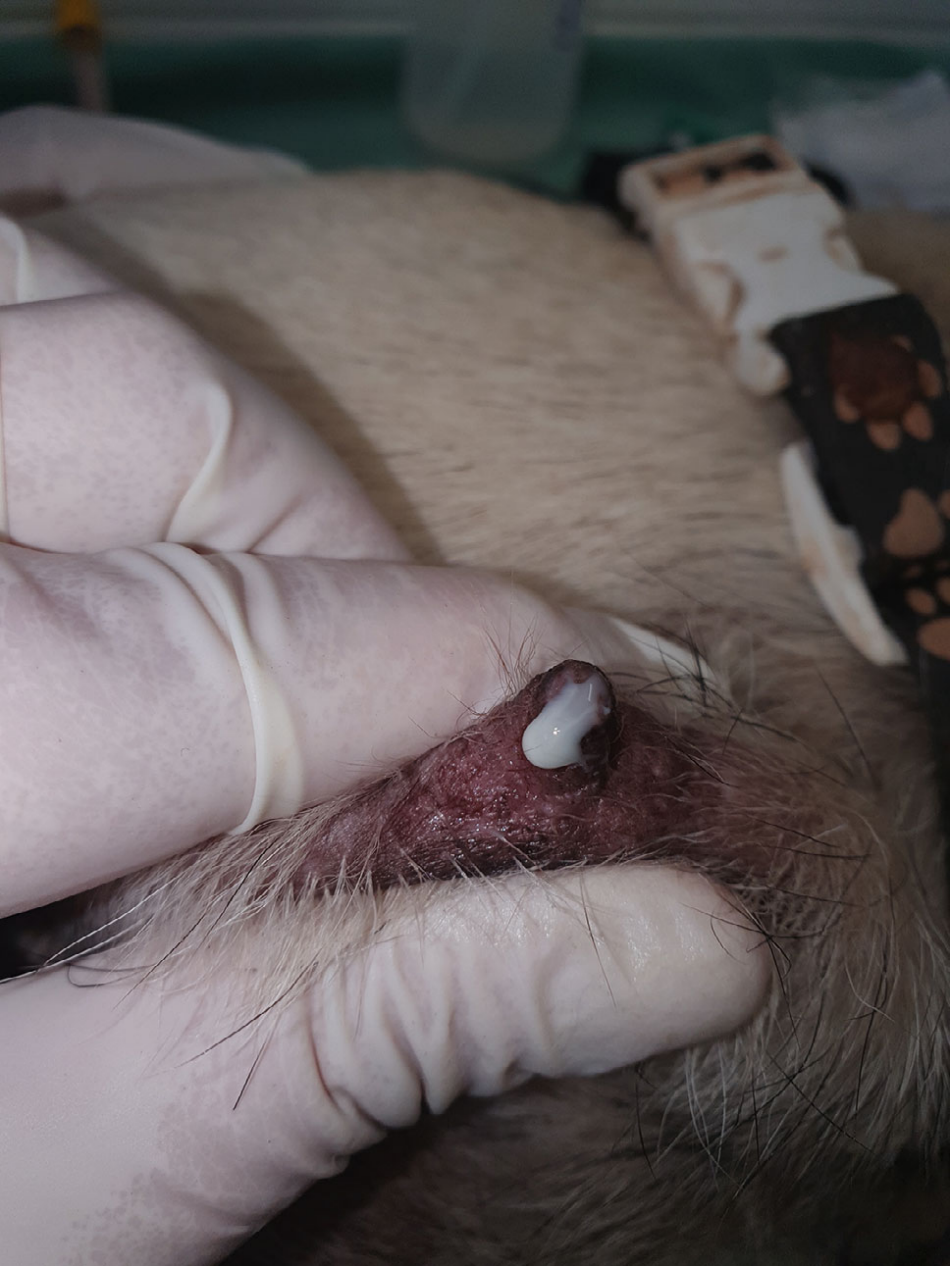
Purulent secretion when expressing milk. Breast milk should not be grayish or dark yellow or contain blood. These changes may be associated with mastitis and the presence of bacteria in milk.

In the maternal clinical examination, the bitch presented vital parameters without changes, normodipsia, normorexia, normuria, and normochesia. When milk was collected, the presence of purulent secretion was observed in all the breasts (Figure 4), with no edema, erythema, or hyperthermia of the mammary glands. Additional tests, such as blood count, cytology, and milk culture with an antibiogram, were requested. The milk sample was cultured on blood agar and MacConkey agar and enriched in brain–heart infusion medium. Antibiograms were performed on Mueller–Hinton agar. The blood count was within normal limits. Cytology revealed the presence of inflammatory cells (neutrophils) in the milk, and microbiological culture revealed the presence of *beta-hemolytic Streptococcus* bacteria, which were sensitive to cephalosporins and penicillins in the antibiogram. Thus, neonatal sepsis caused by toxic milk syndrome likely resulting from maternal clinical mastitis was diagnosed.

The newborns were treated with antibiotic therapy (started before the culture results) with 50 mg/kg ceftriaxone intravenously and subsequently subcutaneously every 12 h for 7 days. The puppies were separated from their mother and started to be fed breast milk with a warm (37°C) milk substitute (Support Milk Dog) in a volume of 3 mL/100 g of weight every 3 h, using a bottle. Water was administered orally between feedings in a volume of 1.5 mL/100 g of weight. Neonatal heating with warm water bags was maintained; stimulation to urinate and defecate with gentle massage of the genitalia, with the aid of moistened cotton, after eating; and daily weight gain, which was monitored with a digital scale. The bitch was treated with amoxicillin with 20 mg/kg potassium clavulanate orally every 12 h for 7 days and with 0.1 mg/kg metergoline every 12 h for 7 days to dry the milk.

After 7 days of treatment, the newborns were clinically healthy, with no signs of infection, blood counts within normal values, and good development (average weight gain of 7% per day). The mother was also clinically healthy. When her breasts were milked, the dog still produced milk, but there was no purulent secretion. A milk sample was collected for culture, and after 48 h, a negative result was obtained. Therefore, it was decided to return the newborns to breastfeeding and maternal care.

## 3. Discussion

Bacterial infections resulting from mastitis and consequent toxic milk syndrome are common in neonatal practice. A study evaluating 113 puppies with sepsis demonstrated that mastitis was the cause of infection in 16% of newborns [5]. Mastitis can occur due to the penetration of pathogens through the mammary ducts and is often facilitated by factors such as trauma, inadequate breastfeeding, and poor hygiene conditions [14]. The risk factors for infection of the mammary glands of the bitch in this report may be related to the fact that the mother has access to the yard and constantly lies on the ground, which increases contact with pathogenic agents, as well as the state of maternal immunosuppression, since the bitch presents physiological temporary immunosuppression during pregnancy, which extends to the lactation period [15].

The signs of infection presented by the neonates in this report, such as diarrhea, body erythema, and omphalitis, are commonly observed in neonates with sepsis [13]. Corporal erythema is due to the systemic vasodilation observed in SIRS; omphalitis can be seen because of vasculitis and SIRS; and diarrhea is a common gastrointestinal disorder in the generalized infection of newborns, especially in cases of toxic milk syndrome, in which bacteria and bacterial toxins are ingested along with milk [11].

Neonate 1 presented the neonatal triad (hypothermia, hypoglycemia, and dehydration). Sick newborns may present apathy and a reduced sucking reflex, leading to failure to ingest milk and progressing to the triad, characterizing the fading puppy syndrome. Newborns are more likely to develop this condition because of the immaturity of the liver, kidney, and thermoregulation systems [13]. Furthermore, the newborn presented with bradycardia, probably secondary to hypothermia and hypoglycemia, which reduced neonatal metabolism and HR [16]. After the patient was stabilized with warming and replacement of glucose, a higher HR was observed.

In cases of mastitis due to *beta-hemolytic Streptococcus* described in the literature, the puppies also presented diarrhea, neonatal triad, body erythema, and omphalitis, demonstrating that these clinical signs are common in patients with toxic milk syndrome [5, 17]. In addition, purulent secretion was also observed in bitches with mastitis caused by this bacterial agent [5]. However, this is the first case report of mastitis and toxic milk syndrome due to *beta-hemolytic Streptococcus* described in Pug dogs.

The blood counts of the neonates in this report revealed leukopenia due to neutropenia and monocytosis, which are changes observed in neonatal sepsis in dogs. A previous study demonstrated the frequency of the most commonly observed changes in the blood count of these patients: 85.8% had leukopenia, 64.6% had neutropenia, 59.3% had lymphopenia, 23% had a left shift (increase in rods), 38.9% had toxic neutrophils, and 54.8% had monocytosis [5]. However, to confirm bacterial infection, performing microbiological culture is also important.

The bacterial agent *beta-hemolytic Streptococcus*, which was isolated from the milk culture of this case, is frequently described in cases of neonatal infection [6, 8, 17]. In a study by [5], this bacterium was the second most isolated bacterium in neonatal sepsis, with an incidence of 12.3%, demonstrating that *beta-hemolytic Streptococcus* can be present in the uterus, vagina, milk, and feces of the mother; in this way, the bacterial infection is often transmitted from the dog to the fetus/neonate during pregnancy, birth, or lactation [8, 18, 19]. Studies and reports have demonstrated that *beta-hemolytic Streptococcus* was associated with clinical and subclinical mastitis in female dogs whose puppies were diagnosed with sepsis. The bacteria were isolated from breast milk, confirming neonatal infection by maternal mastitis [5, 17]. In many cases, subclinical mastitis goes unnoticed, and the litter is observed with fading puppy syndrome. In these cases, bacteriological examination of the milk is essential to exclude mastitis as the underlying cause.

Despite performing a microbiological culture of the milk and confirming mastitis caused by *beta-hemolytic Staphylococcus*, it was not possible to perform a blood culture for bacterial confirmation in the newborns, which is a limitation of this report since it would confirm the etiological link between mastitis and neonatal sepsis.

It is important to perform an antibiogram to determine the sensitivity or resistance of the isolated bacterial agent to the drug chosen for initial treatment and the possible need to replace the antibiotic [20]. The *beta-hemolytic Streptococcus* isolated was sensitive to cephalosporins and penicillins, considered safe antibiotics for the treatment of neonatal infections [6]. Owing to the hepatic and renal immaturity of newborn dogs, their metabolism and clearance capacity are limited; therefore, water‐soluble drugs, such as beta‐lactams, are safer than fat‐soluble drugs are [20, 21].

The treatment of affected newborns must be immediate and primarily involves selecting broad‐spectrum antibiotics, as well as supportive therapy [22]. The puppies were treated with parenteral antibiotic therapy due to the severity of the infection, starting immediately (before the results of the culture and antibiogram) with the drug ceftriaxone, and the bitch was treated with amoxicillin with potassium clavulanate orally, as she had mastitis without systemic infection. Both antibiotics used are broad‐spectrum antibiotics. One study reported survival rates of approximately 79.4% and 69.2% for septic neonates treated with ceftriaxone and amoxicillin with clavulanate, respectively [5]. Thus, the choice of ceftriaxone for this case was based on the description of greater cure rates of newborn dogs with sepsis, compared to other antibiotics [5]. After the results of the culture and observation of the sensitivity of the bacterial agent to the chosen drugs, these were maintained.

Early diagnosis and treatment were essential for the clinical improvement and survival of the puppies in this report. A high mortality rate (approximately 25%) has been reported in newborn dogs with sepsis, mainly due to failure to diagnose, lack of specialized neonatal care, and lack of early treatment [5]. It is important that veterinarians know how to perform clinical evaluations of newborn patients to identify diseases and provide immediate care.

Given that most neonatal infections are associated with maternal infections, it is essential that the mother be thoroughly evaluated during care. Prevention of mastitis can be carried out with appropriate hygiene practices and monitoring during the postpartum period. Guidance to owners and veterinary health professionals on the importance of hygiene and early detection of clinical signs can contribute to reducing the incidence and severity of mastitis and neonatal infections. Adequate prenatal care, clinical evaluations, complementary exams such as bacteriological examination of milk/colostrum, and constant environmental cleaning must be carried out.

## 4. Conclusion

Mastitis is an important source of infection for newborns and should always be investigated in cases of suspected systemic infection in the litter. The bacterial agent *beta-hemolytic Streptococcus* isolated from milk was sensitive to cephalosporins and penicillins in the antibiogram, with neonatal and maternal infections being responsive to ceftriaxone and amoxicillin with clavulanate, respectively. Given that sepsis can have an acute evolution and a high mortality rate, clinical assessment, early diagnosis, and immediate treatment with antibiotics are crucial for newborns′ survival.

**Nomenclature**
SIRS: systemic inflammatory response syndrome
HR: heart rate
RR: respiratory rate
bpm: beats per minute
mpm: movements per minute

## Ethics Statement

The animals described in this report came from a clinical appointment at a veterinary hospital; the report was written after authorization from the animals′ owner through the signed informed consent form.

## Disclosure

All the authors have read and approved the published version of the manuscript.

## Conflicts of Interest

The authors declare no conflicts of interest.

## Author Contributions

Conceptualization, methodology, writing—original draft preparation, writing—review and editing, K.H.N.P.P., J.C.M., K.M.F., G.M.X., D.R.C., F.F.S., and M.L.G.L.

## Funding

The study was supported by Fundação de Amparo à Pesquisa do Estado de São Paulo, 10.13039/501100001807 (2022/10710‐4).

## Data Availability

No datasets were generated or analyzed during the current study.
